# Tomatoes

**Published:** 1882-03

**Authors:** 


### TOMATOES.

WE again remind our readers, that in order to have tomatoes early, seeds should be planted now. Those who have no hot-bed can sow seed in boxes in the house. Small clay pots, or old tin fruit-cans, can be used if more convenient. Sow the seeds quite thickly. When the plants are about two inches high, select the strongest, and transplant to larger pots, placing only one plant in each pot. Tomato plants grown in this way require the same treatment as house plants, and may be kept among them. In May, or as soon as the weather becomes settled, remove the tomato plants to the open ground. In taking them from the pots, the roots must be handled as carefully as possible. If the plants seem weak, they should be protected for a few days from the sun and from chilling winds. If necessary, support the vines by trellises. Families living on small city lots can cultivate two or three tomato plants. The tomato is naturally climbing, and if allowed to run on the pillars of a porch is quite ornamental. It may be utilized to conceal old fences and other unsightly objects. The dark red, smooth tomato, of medium size, is the most desirable variety.

